# Institutionalizing healthcare hackathons to promote diversity in collaboration in medicine

**DOI:** 10.1186/s12909-018-1385-x

**Published:** 2018-11-20

**Authors:** Jason K. Wang, Shivaal K. Roy, Michele Barry, Robert T. Chang, Ami S. Bhatt

**Affiliations:** 1 Mathematical and Computational Science Program, Stanford University, Stanford, CA USA; 2 Department of Computer Science, Stanford University, Stanford, CA USA; 3 Department of Medicine, Stanford University, 269 Campus Drive, Stanford, CA 94305 USA; 4 Center for Innovation in Global Health, Stanford University, Stanford, CA USA; 5 Department of Ophthalmology, Stanford University, Stanford, CA USA; 6 Department of Genetics, Stanford University, 269 Campus Drive, Stanford, CA 94305 USA

**Keywords:** Hackathon, Medical innovation, Medical technology, Interdisciplinary collaboration

## Abstract

**Background:**

Medical students and healthcare professionals can benefit from exposure to cross-disciplinary teamwork and core concepts of medical innovation. Indeed, to address complex challenges in patient care, diversity in collaboration across medicine, engineering, business, and design is critical. However, a limited number of academic institutions have established cross-disciplinary opportunities for students and young professionals within these domains to work collaboratively towards diverse healthcare needs.

**Methods:**

Drawing upon best practices from computer science and engineering, healthcare hackathons bring together interdisciplinary teams of students and professionals to collaborate, brainstorm, and build solutions to unmet clinical needs. Over the course of six months, a committee of 20 undergraduates, medical students, and physician advisors organized Stanford University’s first healthcare hackathon (November 2016). Demographic data from initial applications were supplemented with responses from a post-hackathon survey gauging themes of diversity in collaboration, professional development, interest in medical innovation, and educational value. In designing and evaluating the event, the committee focused on measurable outcomes of diversity across participants (skillset, age, gender, academic degree), ideas (clinical needs), and innovations (projects).

**Results:**

Demographic data (*n* = 587 applicants, *n* = 257 participants) reveal participants across diverse academic backgrounds, age groups, and domains of expertise were in attendance. From 50 clinical needs presented representing 19 academic fields, 40 teams ultimately formed and submitted projects spanning web (*n* = 13) and mobile applications (n = 13), artificial intelligence-based tools (*n* = 6), and medical devices (*n* = 3), among others. In post-hackathon survey responses (*n* = 111), medical students and healthcare professionals alike noted a positive impact on their ability to work in multidisciplinary teams, learn from individuals of different backgrounds, and address complex healthcare challenges.

**Conclusions:**

Healthcare hackathons can encourage diversity across individuals, ideas, and projects to address clinical challenges. By providing an outline of Stanford’s inaugural event, we hope more universities can adopt the healthcare hackathon model to promote diversity in collaboration in medicine.

**Electronic supplementary material:**

The online version of this article (10.1186/s12909-018-1385-x) contains supplementary material, which is available to authorized users.

## Background

To accelerate the development of new technologies that can improve patient care, diversity in collaboration across medicine, engineering, design, and business is critical [1–3]. Yet, there are limited formal opportunities for undergraduate, graduate, and professional school students across these domains to collaborate and engage with challenges in healthcare. Healthcare-themed hackathons have the potential to bridge this gap by promoting diversity in collaboration in healthcare among a diverse demographic of students and young professionals.

Hackathons originated from technology companies as internal events for engineers and computer scientists to collaborate, brainstorm, and build innovative solutions to challenging, company-wide problems in a concentrated period of time [4, 5]. Since then, the hackathon model has migrated into academic institutions, shifting from company-exclusive, computer programming events to broader innovation sessions where technical aspects of engineering merge with nontechnical aspects of design and business modeling. Often, university-wide hackathons are organized as annual events for undergraduate and graduate engineering students to showcase their knowledge, gain familiarity with new technologies, and connect with faculty, laboratories, and resources on campus [6]. In recent years, the hackathon model has extended beyond the traditional high-technology world to tackle domain-specific problems such as education accessibility, water pollution, and veteran support [7]. Healthcare hackathons in particular have become widely adopted across academic institutions and life sciences companies, having first been popularized by MIT’s Hacking Medicine in 2011 [7].

These collaborative events bring together interdisciplinary teams of students and professionals with medical, engineering, business, and design backgrounds to develop solutions for unmet clinical needs while drawing upon best practices from the technology industry [6]. Healthcare hackathons span 2–3 days and follow a loose timeline of events: 1)problem pitching: clinical needs are presented to participants by healthcare professionals, 2) team formation: interdisciplinary teams form around needs presented, 3) hacking: teams brainstorm concepts and develop a prototype and business model to represent their solution, and 4) presentations: teams present their ideas to judges in competition for a set of prizes. Completed projects are expected to outline a solution, such as a software, hardware, or mechanical prototype in combination with a business model.

In emerging healthcare hackathon literature, a prominent emphasis has been placed on market-driven outcomes [7–9]. Silver et al. proposed a framework of measurable outcomes to evaluate the efficacy of healthcare hackathons including the number of trademarks, patents, companies, and commercialized solutions formed as a direct result of the event [8]. Likewise, Olson et al. examined the outcomes of 12 international hackathons hosted between 2012 and 2015, citing new company formation, venture funding raised, and clinical trials conducted as key success metrics [9]. Healthcare hackathons have the potential to accelerate medical innovation by propelling novel solutions to the market as evident from these outcomes studied thus far.

However, Silver’s framework also introduced “skillset diversity,” defined as representation of hackers from a variety of core disciplines and backgrounds [8]. Indeed, in MIT Hacking Medicine’s initial introduction of the healthcare hackathon model, DePasse et al. highlight a key outcome as “a cross-pollination of ideas from diverse thinkers.” [7] However, diversity in collaboration has not yet been thoroughly evaluated. In this study, we investigate the healthcare hackathon model as a tool for promoting diversity among individuals, ideas, and innovations in addressing clinical challenges. We also outline the event planning process for future organizers, highlighting key design choices intended to foster diversity in collaboration.

## Methods

On the weekend of November 5–6, 2016, Stanford University hosted its first healthcare hackathon (“Health++”). At the onset of planning, the committee defined the event’s mission: “to foster collaboration between engineers, designers, entrepreneurs, and healthcare professionals to address unmet clinical needs.” Throughout the process of curating clinical needs, participants, speakers, and sponsors, the committee sought to promote diversity among participants, ideas (clinical needs pitched), and innovations (projects produced) centered on an overarching theme of healthcare affordability in resource-limited settings.

### Planning

Six months prior to the event, a committee of 20 undergraduates, medical students, and faculty advisors from schools of engineering, business, design, and medicine began the planning process for Health++. The committee had no prior hackathon planning experience, although the two undergraduate committee heads had attended previous technology and healthcare-specific hackathons as participants. Major tasks included fundraising $40,000 from academic and corporate sponsors, recruiting opening and closing ceremony speakers, recruiting judges for project evaluations, and event planning logistics (acquisition of venue space, food, beverages, prototyping supplies) (Additional file 1: Appendix 1, event schedule). Two months prior, the committee began advertising to participants and mentors through email and social media. To recruit diverse participants, emails were tailored towards specific domains of expertise. For clinicians, “hacking” could elicit negative connotations. As such, correspondence with clinicians thoroughly defined the goals of a healthcare hackathon and the importance of medical expertise in generating clinical needs. Correspondence with undergraduate engineers emphasized prizes, corporate sponsors, and challenging technical problems. Acceptance emails to participants and mentors were released one month prior. To achieve a 300-attendee target and to gauge attrition rates, all accepted individuals were asked to re-confirm their attendance via email one week before the event.

### Opening ceremony

On Saturday morning, participants checked-in and were given a nametag and colored sticker denoting their domain of expertise: medical, engineering, business, or design. The opening ceremony included a design thinking (a problem-solving methodology centered on the end user’s needs) workshop, keynote presentation providing an overview of successful digital health initiatives, and panel discussion on challenges in healthcare innovation from perspectives of government, academia, and industry. Notably, speakers stressed that addressing clinical challenges would not be a trivial process, requiring sustained effort far beyond the weekend event.

### Problem pitching

Following the opening ceremony, clinical needs were presented in a series of rapid-fire, one-minute pitches. The time limit was strictly enforced to encourage conciseness. Potential problem pitchers submitted need descriptions electronically up to one week before the event to be screened. Needs were evaluated by the hackathon organizers with faculty input based on relevance to the hackathon’s theme, feasibility given resource and time constraints [10], and the pitcher’s ability to actively participant in the hackathon. Departmental and corporate sponsors were allocated slots to pitch needs specific to their academic field or company. To curate problem statements from diverse clinical specialties, the committee publicized the event and engaged with clinicians in-person at departmental grand rounds.

### Team formation

Pitches were numbered to allow participants to track needs that interested them. A meet-and-greet session was hosted in which problem pitchers assigned to tables corresponding to their pitch number interacted with participants. Teams were allowed to self-assemble, with the maximum team size capped at six to ensure an even distribution of skillsets.

### Hacking

From Saturday afternoon onwards, teams ranging from one person to six people were given time to brainstorm, design, and build. Mentors with expertise in software/hardware engineering, design, business, and various clinical specialties were invited to hold office hours to support teams in the development of ideas, prototypes, and business models. Food and beverages were provided free of charge to participants. Sponsors were invited to establish booths in the communal working space to advertise products and recruitment opportunities.

### Project expo

The organizers used the free online platform Devpost to catalog project submissions and facilitate judging. Presentation materials, including presentation slides, demo videos, and business plans, were due via Devpost^9^ by 2:30 PM on the second day, concluding a 26-h 111hacking period. Upon submission to Devpost, teams self-identified sponsor-specific prizes relevant to their idea. A panel of nine grand prize judges evaluated teams based on their presentation of the problem, business feasibility, technical feasibility, solution novelty, and progress made during the hackathon (Additional file 1: Appendix 2, judging criteria). Grand prize judges were selected from academic, technology, and venture capital sectors. Sponsors were invited to judge teams for department or company prizes. Each judge was assigned ten teams in a large auditorium. The organizers facilitated ten rotations of seven minutes each, providing time for a brief pitch and questions. Judges were distributed to guarantee each team be seen by two grand prize judges. The project expo was designed to allow a handful of judges to fairly evaluate a large number of project teams.

### Final presentations

The top eight teams selected by the grand prize panel of nine judges each gave a five-minute presentation to the entire audience, followed by a three-minute question-answer session. $7500 in monetary awards were distributed, including three grand prizes and nine sponsor-specific prizes. To encourage diverse projects, prize criteria favored distinct problem categories (e.g. global health, mental health, etc.) and project modalities (e.g. emphasis on artificial intelligence, process innovation, etc.). Grand prize winners were chosen from the eight finalist teams. All teams were eligible for sponsor-specific prizes. Winners were announced during an awards ceremony, which concluded with a closing keynote discussing commercialization of a product/service in the healthcare ecosystem.

### Post-hackathon survey and analysis

A post-hackathon survey (see Additional file 1: Appendix 3) was developed to gauge participants’ perceptions of the educational and professional development value of Health++. The survey was primarily composed of Likert scale questions, asking respondents to rate the degree to which they agreed with a statement from 0 (strongly disagree) to 6 (strongly agree). Surveys were administered electronically through Qualtrics and collected up to one month post-hackathon. Requests for attendees to complete the survey were made in-person during the closing ceremony and three times via email in the month following.

Survey questions were coded into themes of educational value [4, 13–15], professional development [1, 2], event structure, diversity in collaboration, and interest in medical innovation by the student-faculty committee [1–3]. Summary statistics from Likert responses were computed using R version 3.3.2 (R Foundation for Statistical Computing; Vienna, Austria).

## Results

From 587 applicants, 475 individuals (80.9%) were accepted based on demonstrated interest in medical innovation, academic background, work experience, and domain of expertise. Of 412 accepted individuals who confirmed their attendance via email one week before the event, 257 participants attended (37.6% attrition rate). From 61 needs submitted, 55 were accepted and 50 were presented during the problem pitching session. During the weekend, 40 teams formed and ultimately submitted completed projects. 17 mentors were in attendance.

### Demographics

**Fig. 1:**
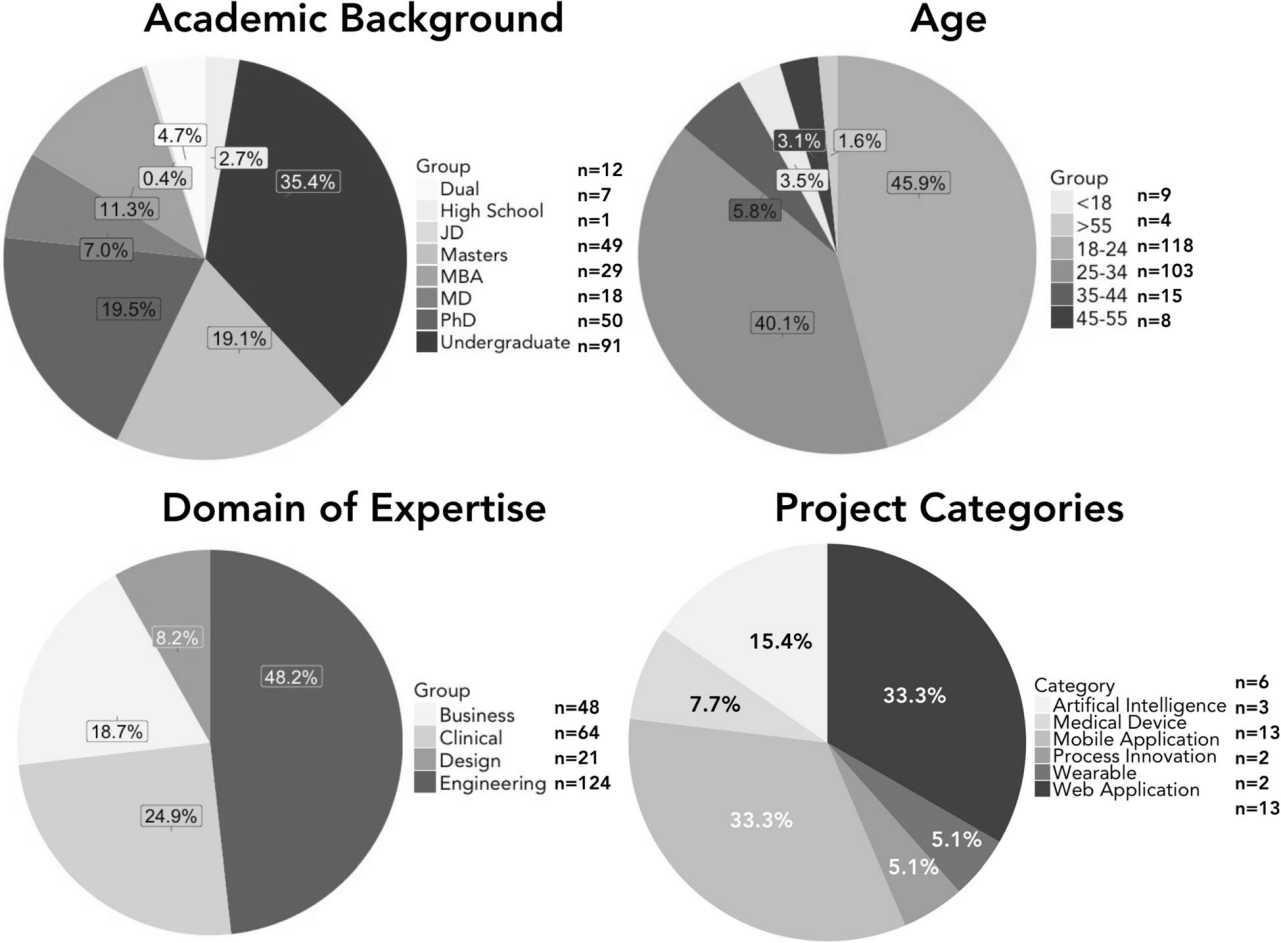
Participants Stratified by Age Group, Domain of Expertise, and Academic Background Alongside Breakdown of Submitted Projects

Demographic data was collected during the application process. The average age of applicants, accepted and confirmed individuals, and participants were 26.3, 26.7, and 25.6 years old, respectively. Notably, physicians (post-MD) in attendance were on average 34.1 years old. In Table 1, applicant, accepted and confirmed, and participant pools are stratified based on educational background and gender. Attrition rates were highest among masters and MD demographics, excluding JD students (*n* = 2). Participants hailed from a diversity of age groups, domains of expertise, and educational backgrounds (Fig. 1). Four domains of expertise were defined: medical (expertise in medicine, biosciences, healthcare services), engineering (expertise in computing, hardware, robotics, artificial intelligence), design (expertise in human-centric thinking, storyboarding, graphic design), and business (expertise in business plan creation, entrepreneurship, management/administration, operations). The predominant demographic was undergraduate engineering students studying computer science, biomedical computation, electrical engineering, or mechanical engineering (Additional file 1: Appendix 7). Business and design specialists were present in the minority.

**Table 1:** Applicant, Accepted and Confirmed, and Participant Pools Stratified By Educational Background and Gender

	Applicant Pool	Accepted & Confirmed Pool	Participant Pool	Attrition Rate^c^
Academic Degree^a^				
High School	10 (1.7%)	9 (2.2%)	7 (2.7%)	22.2%
Undergraduate	220 (37.5%)	135 (32.8%)	91 (35.4%)	32.6%
Masters	131 (22.3%)	110 (26.7%)	49 (19.1%)	55.5%
PhD	93 (15.8%)	70 (17.0%)	50 (19.5%)	28.6%
MD	58 (9.9%)	33 (8.0%)	18 (7%)	45.5%
JD	2 (0.3%)	2 (0.5%)	1 (0.4%)	50.0%
MBA	49 (8.4%)	34 (8.3%)	29 (11.3%)	14.7%
Dual^b^	24 (4.1%)	19 (4.6%)	12 (4.7%)	36.8%
Gender				
Male	328 (55.9%)	320 (55.8%)	148 (57.6%)	53.8%
Female	242 (41.2%)	171 (41.5%)	99 (38.5%)	42.1%
Non-binary	17 (2.9%)	11 (2.7%)	10 (3.9%)	9.1%
Total	587	412	257	37.6%

^a^Academic degree was recorded as a student’s currently enrolled academic program or a professional’s highest level of education

^b^Academic programs categorized under dual degree include MD/PhD, MD/MBA, JD/MBA, and MD/MPH

^c^The attrition rate, defined as the percentage of accepted and confirmed individuals who did not attend the hackathon, is noted for each subgroup. We received correspondence from a minority of accepted applicants (*n* = 11), who upon receiving confirmation emails, stated that they could no longer attend alongside a self-reported reason. Reasons for failed attendance included sickness, unplanned busyness with coursework, familial obligations, conflicting events (e.g. research conferences), and for traveling participants, a lack of funds to support transportation and housing

### Clinical needs

Pitches (n-50) were presented predominantly by clinicians, residents, and medical students (*n* = 41, 82%), with minor representation from business (*n* = 6, 12%) and engineering (*n* = 3, 6%) participants. Clinical needs spanned 19 academic fields with global health (*n* = 8, 16%), healthcare information technology (n = 6, 12%), chronic disease (*n* = 5, 10%), community health (n = 5, 10%), and mental health (n = 5, 10%) being the most represented (Additional file 1: Appendix 10).

### Projects

The three grand prize-winning projects included a smartphone-based application to identify prescription labels for the visually impaired, a leg-wearable measuring gait phase to detect foot drop, and a software tool to identify lower-cost drug and health insurance options. Submitted projects spanned a diversity of author-defined categories (Fig. 1; Additional file 1: Appendix 4, full project list). 39 of 40 project teams made their submission publically viewable on Health++‘s Devpost (Additional File 1: Appendix 5, screenshot) [11].

### Survey results

The post-hackathon survey yielded a 42.7% (*n* = 111) response rate (Additional file 1: Appendix 3). The initial application did not require ethnicity; thus, ethnicity data was acquired only in the post-hackathon survey. Respondents were predominantly White (*n* = 45, 40.5%) and Asian (*n* = 52, 46.8%), with lesser representation from Black or African American (n = 5, 4.5%), Hispanic (*n* = 7, 6.3%), American Indian (n = 1, 0.9%), and Native Hawaiian or Pacific Islander (n = 1, 0.9%). Split by domain of expertise, the respondent pool included 24 medical (21.6%), 49 engineering (44.1%), 12 design (10.8%), and 23 business (20.7%). Split by gender, respondents included 60 males (54.1%), 50 females (45.0%), and 1 non-binary identifying individual (0.9%).Respondent age and academic background were distributed similarly to the participant pool (Additional file 1: Appendix 6). The mean Likert value (MLV) for each statement presented on the post-hackathon survey is shown in Table 2. The post-hackathon survey captured responses from 39 (97.5%) of 40 hackathon teams that submitted completed projects. 34 (87.2%) of 39 teams noted representation from three or more domains of expertise.

**Table 2:** Mean Likert Values for Post-Hackathon Survey Statements

Statement^a^	Mean Likert Value (MLV)^b^				
	Medical (*n* = 24)	Engineering (*n* = 49)	Business (*n* = 23)	Design (*n* = 12)	Total (n = 111)
Educational Value					
I learned about human-centered design	4.25	4.37	3.25	4.44	4.12
I learned about the prototyping process	4.28	4.40	3.30	4.22	4.14
I learned about the components of a business model	4.24	3.89	3.45	3.82	3.88
I learned about the process of entrepreneurship	4.25	3.91	3.35	3.70	3.86
I learned about the healthcare regulatory landscape	3.33	3.84	3.48	4.50	3.71
I learned about the barriers that prevent new innovations from reaching the healthcare market	4.12	4.10	3.77	4.82	4.12
I learned about innovations that are at the forefront of today’s healthcare industry	4.38	4.40	4.27	4.73	4.40
I gained a deeper understanding of the problems facing the healthcare industry	4.25	4.74	4.68	5.00	4.64
After attending health++, I feel much more aware of the cultural context issues in the design of healthcare solutions	4.00	4.54	3.84	4.33	4.27
Professional Development					
The new professional connections I made were valuable	4.96	4.58	3.90	4.45	4.52
For problem pitchers: communicating my need to the health++ audience was a valuable experience/opportunity	5.10	4.80	4.36	3.0	4.73
Event Structure					
Interacting with mentors was beneficial to our team	4.23	3.95	4.31	4.40	4.13
The problem pitching session was valuable in identifying the problems I cared most about	4.62	4.76	4.05	4.36	4.55
Our team was able to quickly identify a specific need or pain point to work on	4.33	4.23	3.55	4.18	4.12
I would have made similar progress without the hackathon	1.19	1.49	1.39	2.00	1.47
I feel that the weekend I spent tackling a validated need has accelerated the development of solutions to improve healthcare	4.57	4.30	3.15	4.22	4.12
In comparison to other outlets and events, health++ is a unique opportunity to learn about healthcare innovation	5.38	5.22	4.85	4.90	5.15
Our team was able to challenge existing paradigms, models, and products that are currently in the healthcare market	5.38	4.74	4.68	5.00	4.23
I intend to continue working on my project and make substantial progress	4.43	3.91	2.67	3.09	3.71
Diversity in Collaboration					
It was valuable working with an interdisciplinary team of diverse backgrounds	5.60	5.38	4.95	5.18	5.33
Our team was able to exchange knowledge and educate each other about our individual areas of expertise	5.38	4.74	4.68	5.00	4.91
After attending health++, I feel more confident in my ability to work with multidisciplinary teams	5.21	4.88	4.35	4.55	4.82
Interest in Medical Innovation					
After attending health++, I feel more confident in my ability to contribute to solving healthcare challenges	4.83	4.78	4.18	4.08	4.59
After attending health++, I feel more inspired to work on problems in healthcare innovation	5.08	4.82	4.59	4.83	4.83
I would like to attend more healthcare hackathons like Health++	5.71	5.52	5.14	5.58	5.50

^a^Statements were designed to gauge participant perception of the educational and professional development value of the healthcare hackathon. Statements also addressed the overall structure and quality of the event and its impact on participant perception of interdisciplinary collaboration and medical innovation

^b^Scores on the Likert scale ranged from 0 to 6 (0 = strongly disagree, 1 = disagree, 2 = somewhat disagree, 3 = neither agree nor disagree, 4 = somewhat agree, 5 = agree, 6 = strongly agree)

Survey respondents agreed most strongly on average with statements gauging diversity in collaboration (average category MLV = 5.02) and interest in medical innovation (average category MLV = 4.97). In particular, respondents scored the value of working in interdisciplinary teams (MLV = 5.33) and their interest in attending future health hackathons (MLV = 5.50) highest. Respondents also agreed strongly with the uniqueness of Health++ in exposing participants to healthcare innovation (MLV = 5.15). Medical domain respondents, comprised of medical students, residents, and healthcare professionals, showed the highest domain-specific MLV across interdisciplinary collaboration (average category MLV = 5.40), professional development (average category MLV = 5.03), and interest in medical innovation (average category MLV = 5.21).

MLVs corresponding to statements under the educational value category ranged from 3.71 to 4.64 (average category MLV = 4.13). In agreement with the statement “our team was able to exchange knowledge and educate each other about [our] individual areas of expertise,” which sought to gauge cross-disciplinary learning, respondents assigned a MLV of 4.91 with medical respondents assigning a domain-specific MLV of 5.38.

## Discussion

Health++ brought together a diverse group of participants with wide-ranging academic backgrounds, age groups, and domains of expertise (Fig. 1). Alongside diverse participation, the event saw similarly diverse clinical needs pitched (Additional file 1: Appendix 10) and projects produced (Fig. 1). Furthermore, survey results show promise that healthcare hackathons can positively impact participant interest in medical innovation and diversity in collaboration, particularly among members of the medical community (Table 2).

Several takeaways can provide value to future healthcare hackathons organizers in promoting diversity across participants, clinical needs, and projects. At Health++, 34 of 37 (87.2%) teams noted representation from three or more domains of expertise, a marked improvement over the same metric (60%) recorded in a longitudinal study evaluating 12 hackathons hosted between 2012 and 2015 [8]. To encourage an even distribution of expertise across project teams, restricting team size to approximately six individuals is suggested. In terms of gender, Health++ favored males (53.8%), although less so than previous events where males comprised between 58 and 75% of recorded participants [8, 9]. To promote gender diversity, invited speakers, judges, and mentors highlighted on the event website and marketing materials should be gender balanced. During early stages of medical innovation, human-centered design and business expertise are as critical as medical expertise [1, 2]. Thus, organizers should aim for a balanced distribution of participants and mentors across all four domains of expertise. To strengthen attendance from design and business domains, health hackathon organizers are encouraged to include design and MBA students in the organizing committee, and use targeted publicity tailored towards specific domains of expertise. Using these methods, relative to the surrounding community represented by Stanford’s enrollment demographics (44.8% engineering, 9.6% medical, 9.1% business) [12], the committee was able to obtain substantial medical and business domain representation (48.2% engineering, 24.9% medical, 18.7% business). In Fig. 1, we observe that only 27 of 257 (10.5%) participants were ages 35 or older, with the average age of post-MD participants at 34.1. This raises the concern that physicians in attendance may be predominantly early-career, a trend also observed in previous healthcare hackathons [8, 9]. To boost attendance from more established physicians, we suggest publicizing through departmental grand rounds and sending personalized emails that highlight less time-intensive opportunities such as problem pitching, mentoring, and part in-person, part remote participation. In accepting applicants, organizers should also account for variable attrition rates to achieve a desired participant pool (Table 1). Many teams discontinue their projects post-hackathon. In fact, survey respondents were indifferent (MLV = 3.71) to the idea of continuing work on their hackathon projects (Table 2). As such, many projects with potential for commercialization are neglected; to encourage post-hackathon development, monetary prizes and investor networking opportunities can be provided to teams that demonstrate substantial progress 3–6 months post-event. Finally, as the problem pitching session largely determines the projects created, organizers should establish a formal vetting process for submitted needs that incorporates faculty review and well-defined evaluation criteria (see Additional file 1: Appendix 8 for suggested criteria). To promote creativity when brainstorming solutions, needs presented should be solution-agnostic [1, 2]. Future organizers are encouraged to publicize their event at departmental grand rounds to solicit needs from diverse clinical specialties.

Silver et al. defined a key goal of healthcare hackathons as providing educational value [8], an outcome evaluated in a number of small-scale, qualitative studies [13, 14]. Indeed, when participants were prompted for motivating reasons they chose to attend Health++, the most frequently cited response (*n* = 94/111, 84.7%) was to “[build] skills and [learn] new things.” Although lecture-based education was not an explicit focus beyond opening ceremony speakers and workshops, we see evidence of cross-disciplinary learning among teams (MLV = 4.91, medical-domain specific MLV = 5.38). Regarding core topics of medical innovation (human-centered design, prototyping, business model development, entrepreneurship, healthcare regulation, etc.), respondents did not feel that the event provided explicit educational value as corresponding MLVs hovering around “somewhat agree” (3.71–4.64). As participants come from a diversity of backgrounds, teaching a common framework of knowledge for medical innovation through a pre-hackathon course or workshop series could improve project quality and provide more concrete educational value to supplement the cross-disciplinary, project-based learning inherent to hackathons. Indeed, healthcare hackathons are not a substitution for comprehensive innovation programs such as Stanford University’s Biodesign Innovation Fellowship [1, 2] or formal courses in medical innovation. Instead, they provide a concentrated period of time (e.g. a weekend) for medical students and healthcare professionals to gain preliminary exposure to multidisciplinary teamwork and the medical innovation process.

The study has several key limitations. The usage of both a pre- and post-hackathon survey would provide a more robust understanding of how participants’ perceptions of medical innovation and interdisciplinary collaboration change as a result of attending healthcare hackathons, and enable the assessment of how such events can increase understanding of core academic topics in medical innovation [3]. Furthermore, the sample size is limited geographically; the majority of participants were students, trainees, staff, or faculty at institutions in Northern California such as UC Berkeley, UCSF, or Stanford (Additional file 1: Appendix 9). Replicating the same survey study at university-based healthcare hackathons across different geographic settings can provide further insight into the value of such events for promoting interdisciplinary education. Lastly, the study evaluates participant perceptions exhibited in the immediate days following the hackathon. Administering a 3- or 6-month post-hackathon assessment can better gauge the long-term impact of healthcare hackathons on aspects including career trajectory.

While prior healthcare hackathons were largely faculty- or institution-driven [8, 9, 13, 14], Health++ was predominantly student-run. Nonetheless, faculty endorsement and funding from university departments were critical in building the event’s reputation among industry sponsors and potential participants during the early stages of planning. Institutionalizing healthcare hackathons by supporting student organizers with consistent (e.g. annual) departmental funding and faculty support can be a worthwhile investment [16, 17]. By providing an outlet for medical students and healthcare professionals to engage in multidisciplinary teamwork and members of the engineering, business, and design communities to direct their talents towards unmet clinical needs, healthcare hackathons can foster a much-needed culture of diversity in collaboration in medicine.

## Conclusions

Healthcare hackathons can promote diversity across people, ideas, and projects to address clinical challenges, and positively impact participants’ perceptions of working in multidisciplinary teams, learning from individuals of different backgrounds, and addressing complex healthcare challenges. By providing an outline of Stanford’s inaugural event, we hope more universities can adopt the healthcare hackathon model to encourage diversity in collaboration in medicine.

## Additional file

Additional file 1: Supplemental Tables, Figures, Questionnaires, and Itineraries 1–10. Additional items highlighting key concepts of planning and evaluating the hackathon event. (DOCX 910 kb)

## Abbreviation

MLV: mean Likert value
