# Supplementary material for: Institutionalizing healthcare hackathons to promote diversity in collaboration in medicine
Source: BMC Med Educ. 2018 Nov 20;18:269. doi: 10.1186/s12909-018-1385-x (PMC6245929; doi:10.1186/s12909-018-1385-x)
Supplement: Supplementary file 1 — Supplemental Tables, Figures, Questionnaires, and Itineraries 1–10. Additional items highlighting key concepts of planning and evaluating the hackathon event. (DOCX 910 kb) [file 12909_2018_1385_MOESM1_ESM.docx]

**Supplemental Digital Appendix**

**Appendix 1.** An itinerary of events for the weekend of Health++.

| **Saturday, November 5** | | **Sunday, November 6** | |
| --- | --- | --- | --- |
| Registration, Breakfast | 8:00 AM | Breakfast | 8:00 AM |
| Welcome from Organizers | 9:00 AM | Lunch | 12:00 PM |
| Design Thinking & Brainstorming 101 | 9:10 AM | Hacking Concludes | 2:30 PM |
| Opening Keynote | 9:30 AM | Project Expo | 3:00 PM |
| Speaker Panel | 9:45 AM | Final Round Presentations: Top 8 Teams | 4:40 PM |
| Problem Pitching | 11:00 AM | Dinner | 6:00 PM |
| Team Formation, Lunch | 12:00 PM | Closing Keynote | 6:30 PM |
| Hacking Begins | 12:30 PM | Prizes & Closing Ceremony | 6:45 PM |
| Dinner | 5:00 PM | Health++ Concludes | 7:30 PM |
| Doors Close | 12:00 AM |  |  |

**Appendix 2: Judging Criteria**

The following is an example of judging criteria drawn from the Health++ Hackathon held on November 5-6, 2016 at Stanford University.

1. Problem Definition: what is the problem and why is it important?

- Ability to articulate targeted pain point(s) around the problem space
- Clearly defined use case that matches articulated pain points
- Potential for impact

2. Technical Feasibility: what are the technical specifications?

- Feasibility of implementation
- Solution provided is technically sound
- Plans for a Beta version are clearly laid out

3. Business Feasibility: how will your solution reach the hands of patients?

- Path to market
- Business model/scalability/distribution

4. Creativity and Newness of the Idea: what solutions exist and why is yours different?

5. Progress During the Hackathon: what did you accomplish this weekend?

- Prototype development stage
- Presentation thoroughness
- Next steps
- Story of progression

**Appendix 3: Post-Hackathon Questionnaire**

1. Age

- <18
- 18-24
- 25-34
- 35-44
- 45-55
- >55

2. Gender

- Male
- Female
- Other: [fill in]

3. Ethnicity

- American Indian

4. What level of education or employment best describes you?

- High School Student
- Undergraduate Student
- Masters Student
- Medical Student
- PhD Student
- MBA Student
- Post-Doctoral
- I have completed schooling and am employed: [fill in] (title, employer)

5. What was your most recent area of study?

6. What was the name of the project you worked on?

7. How many people were on your team (including yourself)?

8. Which role did you play on your team? Check one.

- Medical
- Engineering
- Business
- Design

9. Which role(s) were represented in your team?

- Medical
- Engineering
- Business
- Design
- Other: [fill in]

10. Why did you attend your first medtech hackathon? Check all that apply.

- Networking and connecting to others in the healthcare industry
- Creating a solution to a problem I care about
- Learning about challenges facing the healthcare industry
- Contributing to innovations at the forefront of the healthcare industry
- Being a part of and staying connected to the healthcare innovation community
- Nurturing my inner creativity
- Building skills and learning new things
- Other: [Fill in]

11. Rate from 1 (Strongly Disagree) to 5 (Strongly Agree)

1. The design thinking & brainstorming 101 workshop helped provide a framework for understanding needfinding and ideation
2. The keynote speaker and opening panel were valuable in providing an overview of healthcare innovation as an industry
3. Interacting with mentors was beneficial to our team
4. For problem pitchers: communicating my need to the health++ audience was a valuable experience/opportunity
5. The problem pitching session was valuable in identifying the problems I cared most about
6. Our team was able to quickly identify a specific need or pain point to work on
7. The new professional connections I made were valuable
8. It was valuable working with an interdisciplinary team of diverse backgrounds
9. I learned about human-centered design
10. I learned about the prototyping process
11. I learned about the components of a business model
12. I learned about the process of entrepreneurship
13. I learned about the healthcare regulatory landscape
14. I learned about the barriers that prevent new innovations from reaching the healthcare market
15. I learned about innovations that are at the forefront of today’s healthcare industry
16. I gained a deeper understanding of the problems facing the healthcare industry
17. I feel that the weekend I spent tackling a validated need has accelerated the development of solutions to improve healthcare
18. I would have made similar progress on my project without the hackathon
19. Our team was able to exchange knowledge and educate each other about our individual areas of expertise
20. Our team was able to challenge existing paradigms, models, and products that are currently in the healthcare market
21. In comparison to other outlets and events, health++ is a unique opportunity to learn about healthcare innovation
22. I intend to continue working on my project and make substantial progress
23. After attending health++, I feel more confident in my ability to contribute to solving healthcare challenges
24. After attending health++, I feel more inspired to work on problems in healthcare innovation
25. After attending health++, I feel more confident in my ability to work with multidisciplinary teams
26. After attending health++, I feel much more aware of cultural context issues in the design of healthcare solutions
27. I would like to attend more healthcare hackathons like health++

12. Do you have any specific feedback, recommendations for improvement, and/or reflections about the event(s)?

13. Are there any other resources/programming outside of hackathons that could be provided in the future to help you or your team succeed?

**Appendix 4: Overview of Submitted Projects**

| **Company Name** | **Category** | **Objective** |
| --- | --- | --- |
| Deep Pill Finder | Artificial Intelligence | Deep learning model trained to predict what a medication/pill is based on an image. |
| Foot++ | Medical Device | Medical device to help correct foot drop gait for patients with multiple sclerosis. |
| Conversational Health | Artificial Intelligence | Artificial intelligence-based chatbot that enables patients and providers to call up information on demand for the last mile of care. |
| OnCall | Mobile Application | A mobile application that connects doctors and surgeons in emergency situations to a network of specialists from prestigious institutions to ensure the highest quality of care for all, regardless of geographic location. |
| Jargone | Web Application | Web platform that integrates with existing electronic medical record systems and overlays patients' personal medical records to translate medical jargon into easy-to-digest information. |
| Heart++ | Wearable | Smart watch that uses a heart rate sensor and machine learning to detect heart diseases. |
| Trinity Health | Process Innovation | A business model innovation to reduce maternal death by using an obstetric health insurance model. |
| Dermyx | Artificial Intelligence | Mobile application for rapid detection of skin-based pathologies through a novel computer vision algorithm. |
| PeerMind | Artificial Intelligence | Artificial-intelligence based recommendation engine to connect patients under mental distress with the perfect intervention. |
| MegaHealth | Mobile Application | Comprehensive mobile application for real-time patient booking and patient-delivery (Uber is automatically deployed upon patient booking an appointment). |
| Ready 2 Receive | Web Application | Web-based platform that facilitates direct communication between physicians during hospital transfers. |
| R-NET | Artificial Intelligence | Web-based platform for the structured categorization of medical images using radiology reports; building the next ImageNet (machine-learning based classification) for radiology. |
| SleepWell | Mobile Application | Mobile application that provides reminders and motivation for more sleep. |
| Healthy Data Inc. | Mobile Application | Mobile application to track patient feedback on care. |
| Leaf | Web Application | Web platform that allows for the transfer of patient data among healthcare providers; empowers patients by allowing them to easily access personal health records from different EHRs. |
| Stroke Busters | Process Innovation | A business model innovation to allow for outpatient quality care at home; custom line of rehabilitation equipment that's small, easy to transport, and easy to use. |
| UDDeR | Web Application | A web platform for physicians to update and view distributions of diseases across diverse regions |
| Dance4Healing | Web Application | A low-cost web platform to enable patients and elders to dance with caregivers, other patients, friends and families remotely for home-care. |
| Sherpa | Web Application | An online service that optimizes patient triage in onsite work clinics. |
| Patient+ | Web Application | A web and mobile app to help with the emotional and physical stresses of surgery by helping connect health workers with those in need. |
| FoodChain | Mobile Application | A mobile application that connects local grocery stores to nearby volunteers to make healthy food accessible for all. |
| Pharmassist | Mobile Application | A mobile application and low-cost conductive ink technology that enables visually impaired individuals to use a smartphone to read prescriptions. |
| Clarity Health | Web Application | A website to allow patients to request their medical records from providers and allow to send to new providers. |
| Pallify | Web Application | A web app to improve end of life care through patient education. |
| Affordable Lifestyle Intervention w/ Precision Food | Web Application | A web platform that uses 23andme data to sort through genes and connect relevant genes to appropriate education and provider-mediated interventions. |
| Edge | Mobile Application | A next-generation personal assistant for managing chronic disease in a non-intrusive and accessible manner. |
| RADmosquito | Web Application | A web-based risk advisory dashboard that aggregates data to create a predictive model to enable cost-effective resource allocation and targeted planning for mosquito-borne disease surveillance and control. |
| MoveIt! | Mobile Application | An app to motivate people to move and make lifestyle changes which, in the future, can lead to permanent healthy habits. |
| Savvy: Decreasing Teen Stress by Demystifying | Mobile Application | An app for the personalized development of teens to help reduce stress. |
| Thea | Mobile Application | An app that allows physicians to remotely monitor treatment and progression of amblyopia and strabismus via the patient's smartphone so that patients do not have to make multiple trips to the clinic. |
| BREA: A predictive risk assessment platform | Medical Device | A device that contains a spirometric-like peak flow meter, pulse oximeter, fractional exhaled nitric oxide monitor, and set of microphones to measure lung sound for analysis of inflammation. |
| Benjamin | Mobile Application | An app that patients can use to find lower cost alternative drugs--live in the waiting room with their doctors. |
| LifeWatch | Mobile Application | A smart watch app that encourages good habits by rewarding positive outcomes. |
| WeCare | Web Application | A platform to facilitate the "health coach" model of healthcare by helping people in the community find "health coaches". Health coaches are recruited based on the needs of the community, reflect the community, understand the community, and help mobilize the community to care for itself. |
| EMRWave | Wearable | A wearable that can be used with a secure mobile service to retrieve POLST data. A fast and reliable way for EMS and ER providers to access POLST form data and provide appropriate care. |
| Lamphify | Medical Device | A rapid diagnostic tool for diseases using DNA amplification technologies. |
| CardioHack | Web Application | A tele-monitoring API to help doctors monitor the cardiac condition of their patients. |
| Journal | Mobile Application | Mobile application that allows a patient to keep track of their own personal health record. |
| SafeBridge | Artificial Intelligence | An unbiased third party AI agent powered by a natural language processing software, to encourage intimate and honest disclosure of user’s stories in the privacy of their own home, or anywhere with a mobile device. |

**Appendix 5:** A screenshot of the Health++ Devpost project library. The Devpost platform was used to catalog hackathon project submissions. After the event, the Health++ project library was made publically viewable.^9^


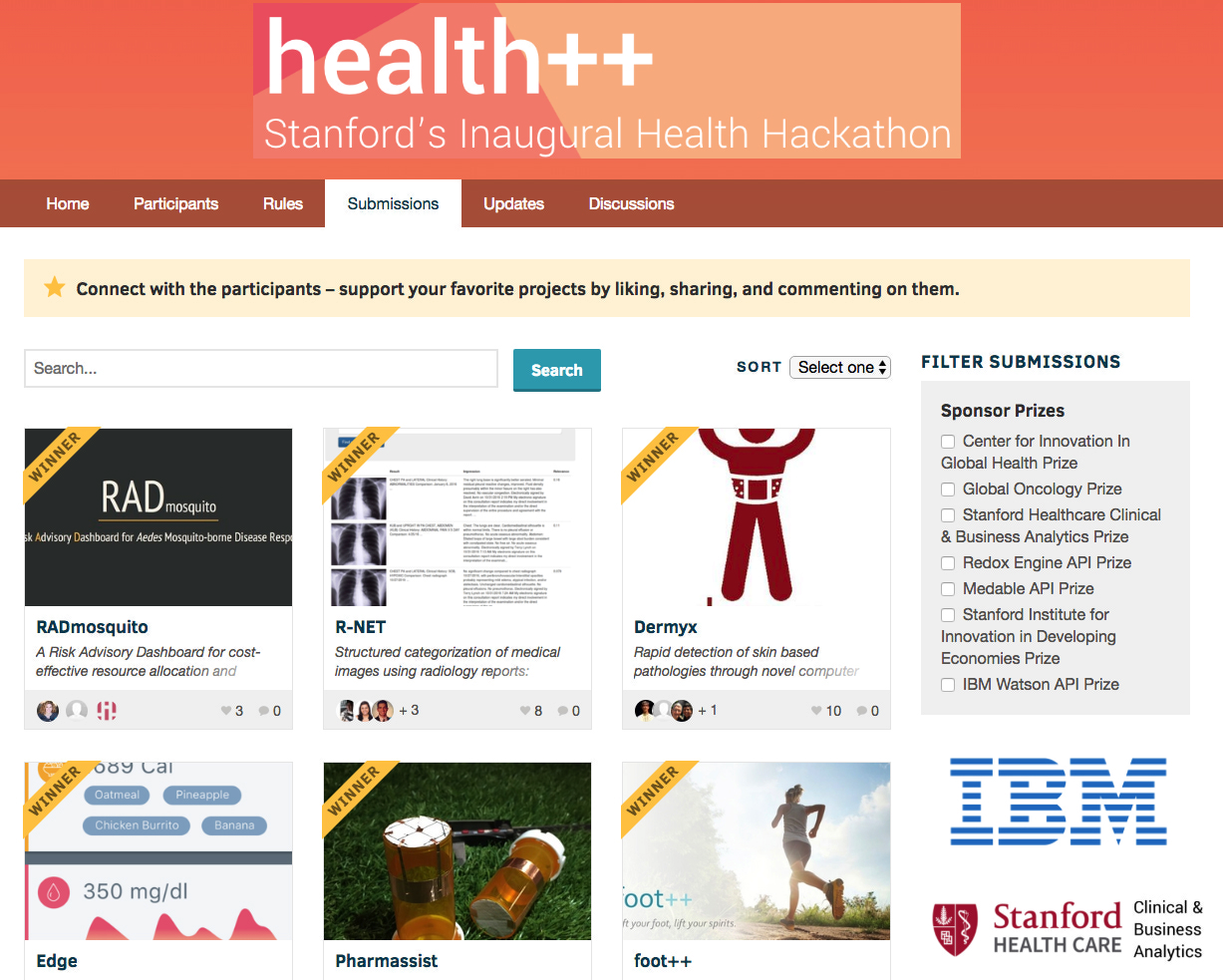


**Appendix 6: Survey Respondents Categorized Based on Educational Background and Age Group**

| **Academic Degree** | |
| --- | --- |
| High School | 5 (4.5%) |
| Undergraduate | 26 (23.4%) |
| Masters | 18 (16.2%) |
| PhD | 11 (9.9%) |
| MD | 4 (3.6%) |
| JD | 0 (0.0%) |
| MBA | 8 (7.2%) |
| Dual | 6 (5.4%) |
| Post-Doctoral | 8 (7.2%) |
| Professional | 25 (22.5%) |
| **Age Group** | |
| <18 | 5 (4.5%) |
| 18-24 | 47 (42.3%) |
| 25-34 | 52 (46.8%) |
| 35-44 | 4 (3.6%) |
| 45-54 | 2 (1.8%) |
| >55 | 1 (0.9%) |
| **Total** | **111** |

**Appendix 7: Breakdown of Undergraduate Participants by Major**

| **Major** | **Count** |
| --- | --- |
| Biomedical Computation | 8 (8.8%) |
| Biology | 3 (3.3%) |
| Statistics | 1 (1.1%) |
| Psychology | 2 (2.2%) |
| Business | 3 (3.3%) |
| Biomechanical Engineering | 2 (2.2%) |
| Electrical Engineering | 5 (5.5%) |
| Industrial Engineering | 1 (1.1%) |
| Economics | 1 (1.1%) |
| N/A | 13 (14.3%) |
| Cognitive Science | 1 (1.1%) |
| Human Biology | 3 (3.3%) |
| Health Policy | 1 (1.1%) |
| Product Design | 2 (2.2%) |
| Mechanical Engineering | 4 (4.4%) |
| Science, Technology, & Society | 2 (2.2%) |
| Computer Science | 28 (30.8%) |
| Bioengineering | 8 (8.8%) |
| Biophysics | 1 (1.1%) |
| Symbolic Systems | 1 (1.1%) |
| Chemical Engineering | 1 (1.1%) |
| **Total** | **91** |

**Appendix 8: Evaluation Criteria for Submitted Clinical Needs**

The following are suggested criteria for student and faculty organizers to consider in evaluating submitted clinical needs:

- - - 1. Relevance to healthcare:
- Would addressing the need improve patient care or healthcare processes?
  - - 1. Relevance to the hackathon’s theme:
- Does the need fit into the overall hackathon theme (e.g. in the case of healthcare affordability, would addressing the need improve healthcare in low-resource settings)?
  - - 1. Feasibility given resource and time constraints:
- Given hardware, software, and human resources available at the hackathon, can meaningful progress be made during the two-day hacking period?
  - - 1. Creativity and newness of the idea:
- Has the need already been addressed by academic research or industry?
  - - 1. Pitcher’s ability to actively participant in the hackathon:
- Can the individual who is pitching the need participate in-full or in-part over in the hackathon itself to provide consistent domain knowledge?

**Appendix 9: Breakdown of Participants by Current or Most Recent Educational Institution**

| **University** | **Count** |
| --- | --- |
| Stanford University | 131 (51.0%) |
| UC Berkeley | 20 (7.8%) |
| UCSF | 11 (4.3%) |
| Other | 95 (37.0%) |
| **Total** | **257** |

| **Academic Field** | **Count** |
| --- | --- |
| Cardiology | 1 (2%) |
| Chronic Disease | 5 (10%) |
| Community Health | 5 (10%) |
| Genomics | 2 (4%) |
| Gerontology | 3 (6%) |
| Global Health | 8 (16%) |
| Healthcare Insurance | 1 (2%) |
| Healthcare IT | 6 (12%) |
| Healthcare Quality | 3 (6%) |
| Maternal Health | 2 (4%) |
| Mental Health | 5 (10%) |
| Oncology | 2 (4%) |
| Ophthalmology | 1 (2%) |
| Orthopedics | 1 (2%) |
| Palliative Care | 1 (2%) |
| Pediatrics | 1 (2%) |
| Radiology | 1 (2%) |
| Sleep Medicine | 1 (2%) |
| Surgery | 1 (2%) |
| **Total** | **50** |

**Appendix 10: Breakdown of Clinical Needs by Academic Field or Clinical Specialty**
